# Feature-enhanced text-inception model for Chinese long text classification

**DOI:** 10.1038/s41598-023-29013-0

**Published:** 2023-02-06

**Authors:** Guo Yang, Yan Jiayu, Xu Dongdong, Guo Zelin, Huan Hai

**Affiliations:** 1 School of Electronic and Information Engineering, Nanjing University of Information Science and Technology, Nanjing, 210044 China; 2 School of Artificial Intelligence, Nanjing University of Information Science and Technology, Nanjing, 210044 China

**Keywords:** Computer science, Software

## Abstract

To solve the problem regarding unbalanced distribution of multi-category Chinese long texts and improve the classification accuracy thereof, a data enhancement method was proposed. Combined with this method, a feature-enhanced text-inception model for Chinese long text classification was proposed. First, the model used a novel text-inception module to extract important shallow features of the text. Meanwhile, the bidirectional gated recurrent unit (Bi-GRU) and the capsule neural network were employed to form a deep feature extraction module to understand the semantic information in the text; K-MaxPooling was then used to reduce the dimension of its shallow and deep features and enhance the overall features. Finally, the Softmax function was used for classification. By comparing the classification effects with a variety of models, the results show that the model can significantly improve the accuracy of long Chinese text classification and has a strong ability to recognize long Chinese text features. The accuracy of the model is 93.97% when applied to an experimental dataset.

## Introduction

Text classification is a basic task in natural language processing. The text classification task can be divided into two subtasks according to the size of the text: short text classification and long text classification. Long text classification has great application value in crime prediction, news classification, and other scenarios. Compared with short texts such as movie reviews and Weibo comments, long texts are more difficult to classify. The length of a long text sample is usually 500–2000 words, the text vector dimension is very large, and there is a problem of semantic sparsity (whereby key information is hidden in a large amount of useless information). Therefore, the key to the long text classification algorithm is whether it can avoid the influences of interference and extract key information.

Currently, a series of pre-trained models, represented by the Bidirectional Encoder Representations from Transformers Model (Bert)^1^, have achieved very high classification accuracy in short texts. In our previous work, by combining BERT with other models, a feature-enhanced Chinese short text classification model was proposed based on a non-equilibrium bidirectional Long Short-Term Memory network^2^. However, the pre-training model has limitations in terms of the length of the input sample. The length of long texts is excessive and the distribution of information therein is too uneven, such that the pre-training model is not easily applied to long text classification. In recent years, the convolutional neural network (CNN)^3^ and recurrent neural network (RNN)^4^ have been widely used in long text classification tasks. CNN can effectively capture local features, while RNN is good at processing sequence information. Long Short-Term Memory (LSTM) and Gate Recursive Unit Network (GRU)^5^, as the derived algorithms of RNN, can avoid the problems such as gradient disappearance and gradient explosion through use of a gate structure. At present, many researchers combine LSTM and GRU with other models to improve the accuracy of text classification^6–8^. Studies have proved that multi-model fusion can improve the ability of the algorithm to capture features.

To enhance the ability of the network to capture key features and realize effective classification of multi-category long texts, a Chinese long text classification model based on feature-enhanced text-inception was developed. First, key semantic features were extracted through the text-inception module. Then, the parallel architecture was used to integrate the capsule neural network and the GRU to enrich the semantic understanding of the text. Finally, the k-MaxPooling layer was used to realize feature aggregation. Through experimental comparison with multiple models, the feature-enhanced text-inception model has achieved good results with respect to effective Chinese long text classification.

This article is structured as follows. The second section introduces the related work. The third section introduces the model and method of this paper. The fourth section contains information about experimental results and data sets. Finally, the fifth section makes a summary of this paper and puts forward the possible direction in the future.

## Related work

An RNN and its variant networks are widely used in text classification due to their powerful sequence-learning capabilities. Compared with traditional RNN and LSTM models, GRU is faster and can also avoid gradient disappearance and gradient explosion, so it is more suitable for long text classification tasks. Yang et al.^9^ proposed a document classification model that combines GRU and hierarchical attention mechanism, which provided a better performance than traditional RNN models. Wang et al.^10^ proposed a sentiment classification model combining a bi-directional GRU model and an attention mechanism: studies have confirmed that bi-directional GRU is better than unidirectional GRU.

There are some problems encountered when RNN networks are used alone: the dimension of the data is too large, the dimensionality reduction operation is complicated, and the effective acquisition of key features cannot be realized. To overcome these problems, researchers have tried to combine CNN with RNN and other structures. Yoon et al.^11^ combined CNN with Bi-LSTM to propose a multi-channel sentiment analysis model, which was used to understand long-distance semantic information and capture key features. Chen et al.^12^ established a sentiment classification model based on CNN, Bi-LSTM, and a conditional random field to achieve three-category sentiment classification. Zhang et al.^13^ proposed a collaborative CNN-LSTM-attention (CCLA) model to learn the semantic and emotional information contained in documents. This model cannot only focus on the global features of the sample, but also capture key local features. Some CNN modules with special architecture or other models can also achieve good results in long text classification. Hinton et al.^14,15^ built a capsule network (a capsule is a group of neurons) running through a “dynamic routing-by-agreement” process to achieve the transfer of parent and child capsules between layers. Aly et al.^16^ proposed the use of a capsule network to implement hierarchical multi-label document classification and used the hierarchical structure of the capsule network to replace the traditional category hierarchical structure. Yang et al.^17^ applied a capsule network in text classification and enhanced the text classification ability by combining this with other models.

In addition, several studies have confirmed that the use of some modules in the feature aggregation stage can enhance the performance of the network and improve the classification accuracy. Shu et al.^18^ mined the LSTM network with K-MaxPooling and used K-MaxPooling for feature aggregation to achieve accurate text classification. You et al.^19^ developed an extreme multi-label text classification model based on Bi-LSTM and multi-label attention mechanisms. Liu et al.^20^ combined the attention mechanism, Bi-LSTM, and a convolutional layer text classification model, which is similar to the architecture of Zhang^13^, but shows reduced spatio-temporal complexity. Szegedy et al.^21^ proposed an inception module to be applied in a computer vision scenario; this could increase the depth and width of the network while avoiding a substantial increase in the amount of calculation. Basha et al. ^25^ compared the effects of different feature selection techniques on text classification accuracy. The analyzed experimental results show that Naïve Bayes algorithm gives more accuracy in many cases i.e. with many feature selection techniques and K-Nearest Neighbor classifier works well only in the cases, when the feature selection techniques either Information Gain (IG) or Mutual Information (MI). To improve the accuracy of long text classification of Chinese news, Chen et al.^26^ propose a BERT-based local feature convolutional network (LFCN) model including four novel modules. Liang et al.^27^ gave an improved ensemble model for Chinese text classification based on CNN and RNN structure. Guo et al.^28^ propose a text classification method based on the Convolutional and Bi-LSTM Model (CBM), which can extract both the local shallow semantic features and the global deep semantic features. Liu et al.^29^ combined Chinese syntactic dependency tree with graph convolution and proposed a new sentiment classification model (dependent tree graph convolution Network, DTGCN), which improved the performance of sentiment classification in this paper. Based on the above work, in the present work a feature-enhanced text-inception model for Chinese long text classification is proposed.

## Methods

**Figure 1:**
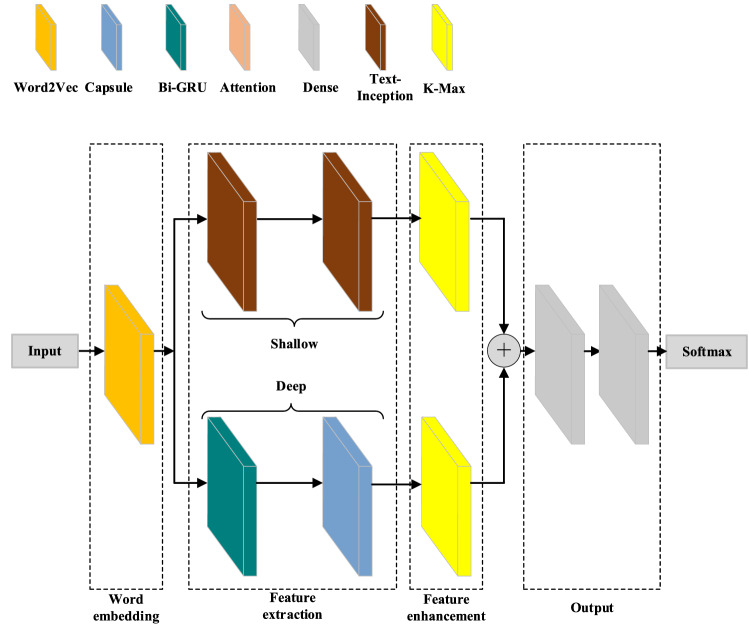
The overall framework of the feature-enhanced Text-Inception model for Chinese long text classification.

The Chinese long text classification model based on feature-enhanced text-inception consists of four parts: a word embedding layer, a feature extraction layer, a feature enhancement layer, and output layer. First, the original text is pre-processed and word embedding layer is put to vectorize the original text. Second, in the feature extraction layer, the text is understood by the shallow key feature extraction module and the deep semantic understanding module. Third, deep and shallow features are fused after feature enhancement by K-MaxPooling. Finally, text classification is implemented in the output layer. The overall framework of the model is illustrated in Fig. 1.

### Data preprocessing and enhancement

**Figure 2:**
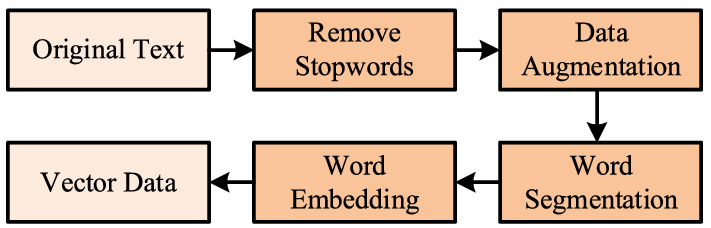
Data pre-processing and data enhancement procedure.

The main function of the word embedding layer is to map the original corpus into a vector form, but the original corpus needs to be pre-processed before embedding. The pre-processing procedure is shown in Fig. 2.

First, useless characters such as punctuation marks and stop words are removed from the original text; because the character length of stop words such as punctuation marks is usually 1, all characters with a length of 1 are removed from the original text. Because the dataset used in the present research contains many categories, and the number of samples in each category is unevenly distributed, it is easy to ignore small sample categories in the learning stage, which leads to poor overall text classification. Therefore, after counting the number of samples, we copy the categories with smaller sample sizes and add them to the original dataset to increase the feature strength of the small sample categories and enhance the learning effect thereof. After pre-processing, the Jieba tool is used to segment the original text, then Word2vec^22^ is employed to vectorize the original text, and finally the vector data are output.

The data enhancement method is as expressed by Eq. (1):

1 $${\{y}_{1},{y}_{2},\dots ,{y}_{n+km}\}=\{{x}_{1},{x}_{2},\dots ,{x}_{n}\}+k\{{x}_{1},{x}_{2},\dots ,{x}_{m}\}.$$

$\{{x}_{1},{x}_{2},\dots \dots ,{x}_{n}\}$ represent all samples in the original dataset. $\{{x}_{1},{x}_{2},\dots \dots ,{x}_{m}\}$ represent categories with small sample sizes. Herein, a category with a sample size of less than 5000 is defined as a small category. We copy all the samples of the small category $k$ times and add them to the original data to obtain new data ${\{y}_{1},{y}_{2},\dots ,{y}_{n+km}\}$ after data enhancement. Experiments prove that this method can effectively improve the effect of the model. Experiments prove that this method can improve the efficacy of the model. The experimental results are displayed in Table 1.

**Table 1:** Experiments on the effectiveness of data enhancement methods.

K	Precision	F1-score	Recall
K = 0	0.9366	0.8071	0.7091
K = 1	0.9371	0.8133	0.7184
K = 2	0.9397	0.8217	0.7300
K = 3	0.9354	0.8108	0.7155

### Feature extraction

Text feature extraction methods are generally divided into two types, shallow key feature extraction based on CNN and deep semantic understanding based on RNN. In short text classification, due to the smaller solution space, both methods can achieve good results, however, in long text classification, the effect of using shallow features or deep semantics alone is not ideal due to the large sample size and scattered feature distribution. Therefore, the fusion of shallow features and deep semantics is used in the present research. To overcome the problem posed by the computational complexity of traditional CNNs (and their insufficient depth), a two-layer text-inception module is used to implement shallow feature extraction. At the same time, the GRU network is combined with a capsule neural network to achieve a deeper level of semantic understanding. The overall structure is shown in Fig. 1.

**Figure 3:**
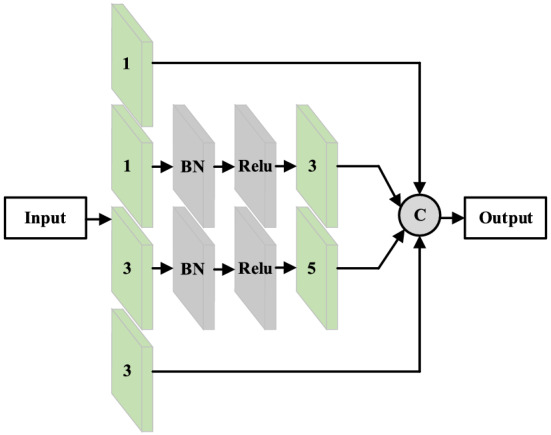
The structure of the Text-Inception module.

Shallow feature extraction module. In the model, two layers of text-inception modules (in series) are combined into a shallow feature extraction module. The architecture of the text-inception module is shown in Fig. 3.

The core idea of text-inception is to increase the depth and width of the network without greatly expanding the amount of calculation while extending the field of experience of the network in terms of salient features and improve the expressive ability of the network. For this reason, 1 × 1 and 1 × 3 convolution kernels are used in the first layer. The smaller convolution kernels ensure that the key features are not omitted, but some features require lengthy correlation, and the smaller convolution kernels cannot learn such features. Therefore, based on the first layer, we add 1 × 3 and 1 × 5 convolution kernels to increase the receptive field of the network and ensure that long-distance features are not omitted. In addition, to avoid gradient disappearance and accelerate convergence of the network, we add batch normalization^23^ layers and ReLU layers between the two convolution layers. The main function of the BN layer is to find a linear and non-linear equilibrium within the network, which cannot only realize the strong expression ability of non-linearity, but also avoid the problem of slow network convergence caused by non-linear saturation. The calculation process enshrined in the text-inception module is given by Eqs. (2) to (6):

2 $${y}_{1}={F}^{1}\left(x,{W}^{1}\right),$$

3 $${y}_{2}={F}^{3}\{\left[{F}^{1}\left(x,{W}^{1}\right),{W}^{3}\right]+{B}_{bn+relu},$$

4 $${y}_{3}={F}^{5}\{\left[{F}^{3}\left(x,{W}^{3}\right),{W}^{5}\right]+{B}_{bn+relu},$$

5 $${y}_{4}={F}^{3}\left(x,{W}^{3}\right),$$

6 $$y=Concat\left\{{y}_{1},{y}_{2},{y}_{3},{y}_{4}\right\},$$

where $x$ and $y$ are the input and output of the text-inception module respectively. ${y}_{1}$, ${y}_{2}$, ${y}_{3}$, and ${y}_{4}$ are the output of each branch, respectively; ${F}^{1}$, ${F}^{3}$, and ${F}^{5}$ are the convolution process when the convolution kernel is 1, 3, and 5, respectively; ${W}^{1}$, ${W}^{3}$, and ${W}^{5}$ are coefficient matrices when the convolution kernel is 1, 3, and 5, respectively; ${B}_{bn+relu}$ is the correction applied to the BN layer and Relu activation function to the convolution process. After all calculations are completed, the output results are input to the next module of the network.

Deep semantic understanding module. RNN is widely used in text classification due to its powerful understanding of time series input. Currently, the main RNNs used are LSTM and GRU, however, due to the high dimension of long text samples, the network requires onerous amounts of computation, while GRU reduces the gate structure of LSTM by one, which greatly improves the computational efficiency without a significant loss of precision. In addition, the bidirectional GRU (Bi-GRU) can realize the understanding of the semantics from the front and back to the two directions compared with the one-way GRU. On the whole, the Bi-GRU network is used as the basis of the deep semantic understanding module. The equation governing use of this Bi-GRU is as follows:

7 $${z}_{t}=\sigma \left({W}_{z}\cdot \left[{h}_{t-1},{x}_{t}\right]\right),$$

8 $${r}_{t}=\sigma \left({W}_{r}\cdot \left[{h}_{t-1},{x}_{t}\right]\right),$$

9 $${h}_{t}^{\sim }=tanh\left(W\cdot \left[{r}_{t}*{h}_{t-1},{x}_{t}\right]\right),$$

10 $${h}_{t}=\left(1-{z}_{t}\right)*{h}_{t-1}+{z}_{t}*{h}_{t}^{\sim },$$

where ${z}_{t}$ is the iterative formula for updating the gate; ${r}_{t}$ represents the iterative formula used when resetting the gate. The inputs are both the output of the previous time slot ${h}_{t-1}$ and the input of this time slot ${x}_{t}$; ${h}_{t}^{\sim }$ is the current unit state. This determines the degree of retention of information from the current and previous time steps through the results of updating and resetting gates; ${h}_{t}$ is an output gate that determines the output at the current time.

However, Bi-GRU learns the correlation of each sequence of the input signal, which weakens the key information to a certain extent. For this reason, a capsule neural network after Bi-GRU is added to integrate the output features of Bi-GRU into capsule feature blocks. These feature blocks contain different feature intensities. Dynamic routing protocol is adopted to retain the high-strength feature capsules on the premise of keeping the spatial information unchanged the further to refine the semantic information.

### Feature enhancement module

**Figure 4:**
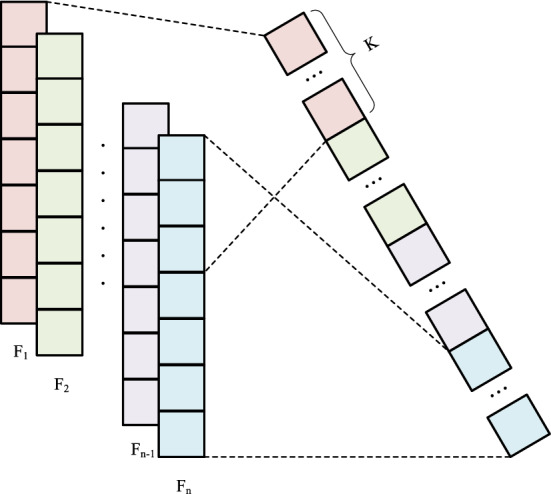
K-MaxPooling schematic diagram.

The sample length of a long text is excessive, thereby reducing the training efficiency. If the dimensionality of the classification end is too large, the classification accuracy will be affected. To solve this problem, the maximum pooling method is often used to reduce the dimensionality of the network. However, there are two obvious problems with traditional max-pooling algorithms: max-pooling only retains a maximum value of each group of eigenvalues, so we only get a maximum value and no positional information about where this maximum value appears; secondly, some strong features will appear multiple times in a set of feature values, and the maximum pooling only retains one, which will cause the feature strength information of this feature to be lost. Due to the above reasons, the k-MaxPooling method is used to reduce the dimensions of the high-dimensional features output by the previous network and the feature information is retained to the maximum extent. A schematic representation of the K-MaxPooling process is shown in Fig. 4.

K-MaxPooling is improved based on traditional maximum pooling. It preserves the largest first K values in a set of eigenvalues and arranges them according to the original sequence of these eigenvalues. K-MaxPooling can retain the strength information of features to the maximum extent, while reasonable selection of the K value can eliminate weak features to a certain extent and retain positional information. Herein, the K value is set to 8.

## Results and discussion

### Datasets and experimental equipment

The main dataset used in this study comprises criminal case description discourses from China judgements (an on-line resource). This dataset^24^ contains 154,592 documents for training, 17,131 documents for validation, and 34,720 documents for testing. There are 202 categories in this dataset, and the distribution of each category is unbalanced. The sample size of the largest category is hundreds of times that of the smallest category, making classification of this dataset is very difficult.

In addition, the other two Chinese datasets are utilized to verify the generalization ability of the feature-enhanced text-inception model for Chinese long text items. The first is the dataset of Daguan Cup Chinese Text Classification Challenge, which consists of 200,000 pieces of data across 20 categories (each sample contains more than 700 words on average). The second is the THUCNews Chinese text classification dataset provided by the Natural Language Processing Laboratory of Tsinghua University, which contains 740,000 pieces of data across 14 categories.

To verify the ability of the model to process non-Chinese texts, we use some datasets in other languages. The first is the German news dataset, which has 10,273 pieces of data across nine categories; the second is the Yelp-5 hotel star rating dataset, which contains English text and consists of five categories, including 130,404 items of training data and 10,056 items of test data.

This experiment was run on a high-performance computer equipped with a Tesla T4 graphics card and 32 Gb memory, using the Keras development framework, and Python (Version 3.6).

### Experimental results and analysis

To verify the effect of the feature-enhanced text-inception model for Chinese long-text classification, multiple experiments are undertaken. The first experiment can verify the effect of data enhancement on the model; the second validates the effect of each module of the model; the third verifies the classification effect of the model by comparing it with other methods as applied across multiple datasets.

Precision and F1-score are used as evaluation indices. Since the sample distribution of each category of the dataset is non-uniform, precision mainly reflects the classification effect of a large sample category. The F1-score represents the classification accuracy of each category, so the higher the F1 score, the better the overall classification effect of the model and the higher the attention to small categories.

**Figure 5:**
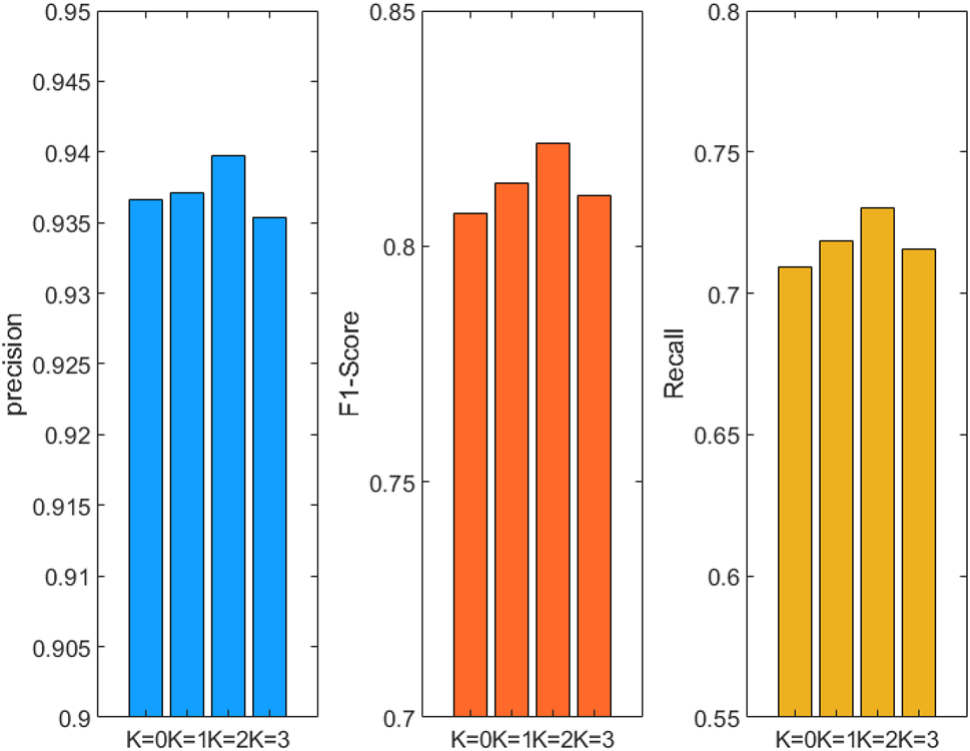
Visualization results of data enhancement methods.

Experiments on the effectiveness of data enhancement methods. To verify whether the proposed data augmentation method can increase the network’s attention to small sample categories, the following experiments are conducted on the CAIL2018 dataset. In the experiment, the K value represents the replication multiple of the small sample category, where K = 0 denotes the use of original data without data enhancement. The experimental results are displayed in Table 1 and Fig. 5.

Through analysis of the data, the enhancement effect of the model is found to be optimized at K = 2. Compared with examples run without data enhancement, the precision is increased by 0.31% and the F1-score is increased by 1.46%. This indicates that the proposed data enhancement method can enhance the attention of the model to small categories of samples. However, when K = 3, the overall precision decreases compared with the original data. This indicates that when the value of K is too large, the number of samples in the original small sample category exceeds that of some large samples, which changes the original category feature distribution and leads to the decline in overall recognition accuracy. Therefore, K is set to 2 in subsequent experiments.

It should be noted that the K value of 2 is only applicable to the CAIL2018 dataset due to the different sample distributions of different datasets. The sample distribution of other experimental datasets referred to in this article is more even and there are fewer categories therein, so this data enhancement method is not used.

**Figure 6:**
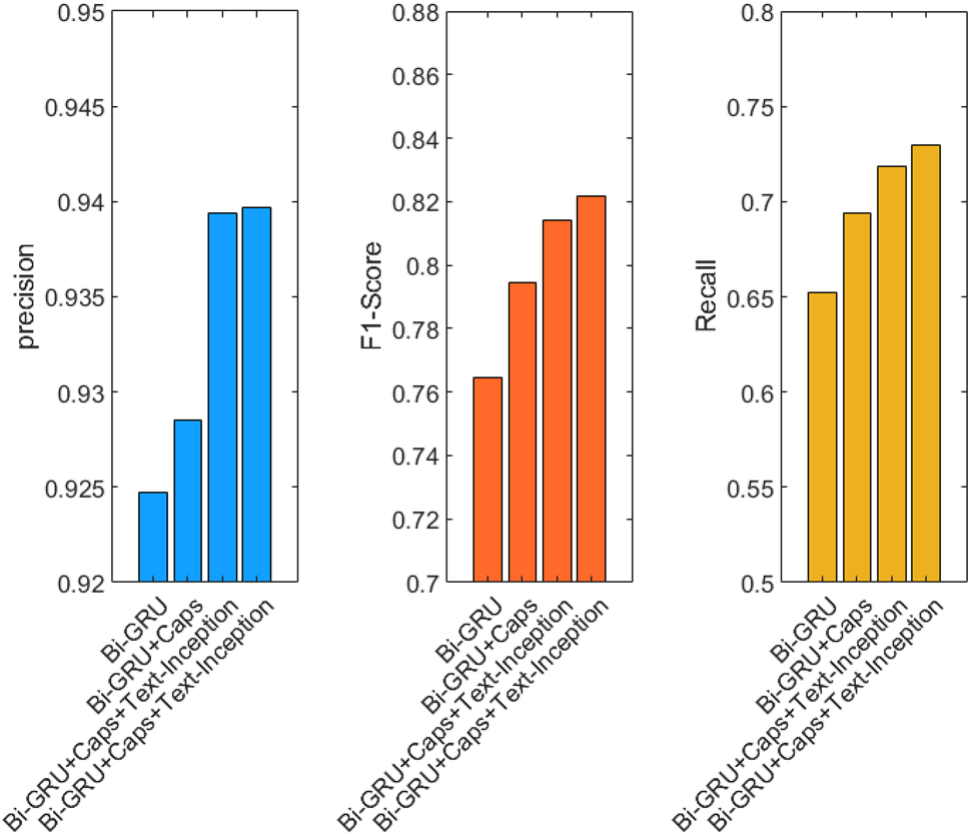
Visual results of module effectiveness.

Validity experiment of each module. To verify the effectiveness of each component of the feature-enhanced text-inception model, each module is added to the basic model Bi-GRU. This set of experiments are also conducted based on the CAIL2018 dataset, and the experimental results are summarized in Table 2 and Fig. 6.

**Table 2:** Validity experiment of each module.

Model	Precision	F1-score	Recall
Bi-GRU	0.9247	0.7647	0.6519
Bi-GRU + Caps	0.9285	0.7944	0.6941
Bi-GRU + Caps + Text-Inception	0.9394	0.8142	0.7184
Bi-GRU + Caps + Text-Inception + K-Max	0.9397	0.8217	0.7300

First, the output of Bi-GRU is connected to the capsule neural network to form a deep semantic understanding module. Compared with the basic model, precision is increased by 0.38%, and the F1-score is increased by 2.97%. This result shows that the capsule neural network can improve the attention of the network to the small sample under the premise of the overall classification accuracy remaining stable.

Thereafter, we concatenate two layers of text-inception to form a shallow feature extraction module and fuse it with a deep semantic understanding module. Compared with the deep semantic understanding module, the precision and F1-score are increased by 1.09% and 1.98%, proving that the shallow feature extraction module can enhance the network’s understanding of semantics by extracting key features and improving the overall accuracy, thus the F1-score of the network is much improved.

Finally, a K-MaxPooling layer is added to the shallow feature extraction and deep semantic understanding modules respectively to reduce dimensionality and conduct feature aggregation before classification. This increases the F1-score by 0.65% without loss of overall accuracy, suggesting that feature aggregation using K-MaxPooling can enhance the features of small sample categories and improve the network’s attention to small feature categories.

Holistic model validity experiment. To verify the overall performance of the feature-enhanced text-inception, the model is compared with other models in multiple datasets. The experimental results are summarized in Table 3.

**Table 3:** Comparison results with other models on the CAIL2018 dataset.

Model	Precision	F1-score	Recall
Bi-GRU	0.9247	0.7647	0.6519
Bi-LSTM	0.9315	0.8128	0.7209
Capsule	0.9334	0.8018	0.7027
VDCNN	0.9277	0.8209	0.7262
Text-inception	0.9362	0.8186	0.7272
Text-CNN	0.9356	0.8172	0.7254
Resnet	0.9349	0.8194	0.7293
The proposed model	0.9397	0.8217	0.7300

On the CAIL2018 dataset, our model achieves the best classification effect: precision reaches 0.9397 and the F1-score reaches 0.8217. Compared with the basic BI-GRU network, our model improves the precision by 1.5% and F1-score by 5.7%. Compared with other models, there are other improvements: feature-enhanced text-inception can better handle the multi-category long text classification task, and pays more attention to small sample categories.

To verify the generalization ability of the feature-enhanced text-inception model, the model is compared with other models on two Chinese datasets: the Daguan dataset and THUCNews. In addition, to verify the classification ability of the model on non-Chinese datasets, our model is compared with other models on the German news dataset and the Yelp-5 hotel star dataset. The experimental results show that the feature-enhanced text-inception model can still achieve the best classification effect. The experimental results are summarized in Table 4.

**Table 4:** Experiment results on other datasets.

Model	Daguan dataset		
	Precision	F1-score	Recall
Bi-GRU	0.7782	0.7642	0.7507
Bi-LSTM	0.7845	0.7812	0.7679
Capsule	0.7924	0.7797	0.7674
VDCNN	0.7852	0.7748	0.7647
Text-inception	0.7947	0.7830	0.7716
Text-CNN	0.7848	0.7825	0.7702
Resnet	0.7866	0.7804	0.7743
The proposed model	0.7973	0.7860	0.7750

Model	THUCNews		
	Precision	F1-score	Recall
Bi-GRU	0.9453	0.9398	0.9344
Bi-LSTM	0.9481	0.9431	0.9352
Capsule	0.9406	0.9373	0.9340
VDCNN	0.9461	0.9371	0.9223
Text-inception	0.9479	0.9422	0.9366
Text-CNN	0.9401	0.9265	0.9133
Resnet	0.9423	0.9321	0.9221
The proposed model	0.9514	0.9442	0.9371

Model	German News Dataset		
	Precision	F1-score	Recall
Bi-GRU	0.7661	0.7288	0.6950
Bi-LSTM	0.7673	0.7101	0.6608
Capsule	0.8089	0.7822	0.7572
VDCNN	0.8128	0.8002	0.7880
Text-inception	0.7412	0.7212	0.7023
Text-CNN	0.8077	0.7880	0.7692
Resnet	0.7214	0.6831	0.6487
The proposed model	0.8141	0.8036	0.7934

Model	Yelp-5 dataset		
	Precision	F1-score	Recall
Bi-GRU	0.5693	0.5532	0.5380
Bi-LSTM	0.5705	0.5582	0.5464
Capsule	0.5607	0.5473	0.5345
VDCNN	0.5647	0.5441	0.5250
Text-inception	0.5643	0.5480	0.5326
Text-CNN	0.5625	0.5428	0.5244
Resnet	0.5618	0.5422	0.5239
The proposed model	0.5707	0.5605	0.5507

On the Daguan dataset, the precision reaches 0.7973 and the F1-score reaches 0.7860. This result indicates that weak semantic information can be better processed by the fusion of shallow and deep features. The Daguan dataset contains desensitized data, the semantic information therein is fuzzy and weakened, making it difficult to achieve good classification effect on this dataset.

On the THUCNews dataset, the precision reaches 0.9514, and the F1-score reaches 0.9442. The overall category distribution across the THCNews dataset is relatively balanced, so the precision of the present experimental results is not much different from the results embodied by the F1-score. On relatively simple datasets, our model can still achieve good results, but the overall classification accuracy is not much improved compared with other datasets. This indicates that our model is better at processing complex and multi-category data and has little advantage in processing data with more uniform feature distribution and more balanced categories.

On the German news dataset, our model can achieve the best classification effect, with a precision reaching 0.8141 and an F1-score reaching 0.8039. Since the overall sample size of the German dataset is small and the relevant features are not obvious enough, the overall classification accuracy is not high enough, but compared with the Resnet model with the lowest accuracy, our model still improves the precision by 9.27%.

On the Yelp-5 dataset, the precision of the model reaches 0.5705, and the F1-score reaches 0.5605. Since the Yelp-5 dataset is a star-rating dataset, the corresponding relationship between the sample and the category is insignificant, so the classification results on each model are not ideal. Our model has the best effect among all tested models, but the improvement in precision is small, while the improvement in F1-score is larger. This proves that our model can enhance the attention of the network to the small sample categories, thus improving the overall classification effect of the network.

## Conclusions

A feature-enhanced text-inception model was proposed for Chinese long text classification. First, a novel text-inception architecture was used to form a shallow feature extraction module. The shallow features were then fused with the deep features extracted by Bi-GRU and capsule neural network. Then, K-MaxPooling was used to achieve dimension reduction, feature fusion, and enhancement. Finally, the desired classification was realized. In addition, a data-enhancement method was proposed for CAIL2018 dataset to enhance small sample categories and preliminarily deal with the problem of unbalanced class distribution in multi-category long texts. Through experimental verification, the combination of the data enhancement method and the feature-enhanced text-inception model can improve the network’s attention to small sample categories, solve the problem of category imbalance to a certain extent, and realize the effective classification of multiple categories of long text.

There remain some unresolved issues: there are some very small sample categories in the CAIL2018 dataset (the feature strength of these categories remains too low after data enhancement). How to address this part of the category warrants further investigation.

## Data Availability

The XML data used to support the findings of this study are included within the article. Previously reported XML data are available at https://github.com/china-ai-law-challenge/CAIL2018. These prior studies (and datasets) are cited at relevant places within the text as references 24.
